# Supplementary material for: Bifunctional MoS2/Al2O3-Zeolite Catalysts in the Hydroprocessing of Methyl Palmitate
Source: Int J Mol Sci. 2023 Oct 3;24(19):14863. doi: 10.3390/ijms241914863 (PMC10573751; doi:10.3390/ijms241914863)
Supplement: Supplementary file 1 [file ijms-24-14863-s001.zip › ijms-2640670-supplementary.pdf]

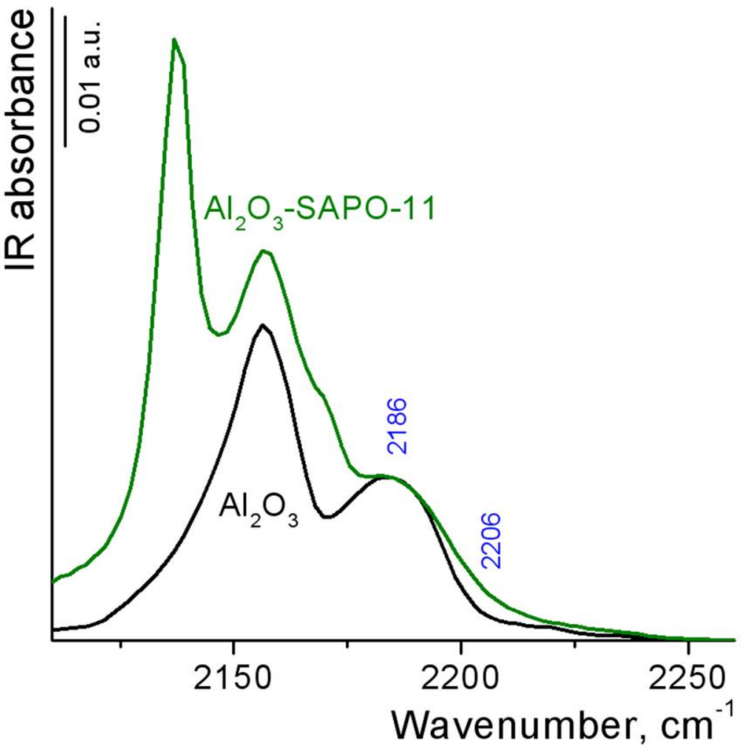

Figure S1. FTIR spectra of CO adsorbed on pure Al_2O_3 and Al_2O_3-SAPO-11 supports. Equilibrium CO pressure is 5 mbar at liquid nitrogen temperature. Spectra normalized to the alumina content.

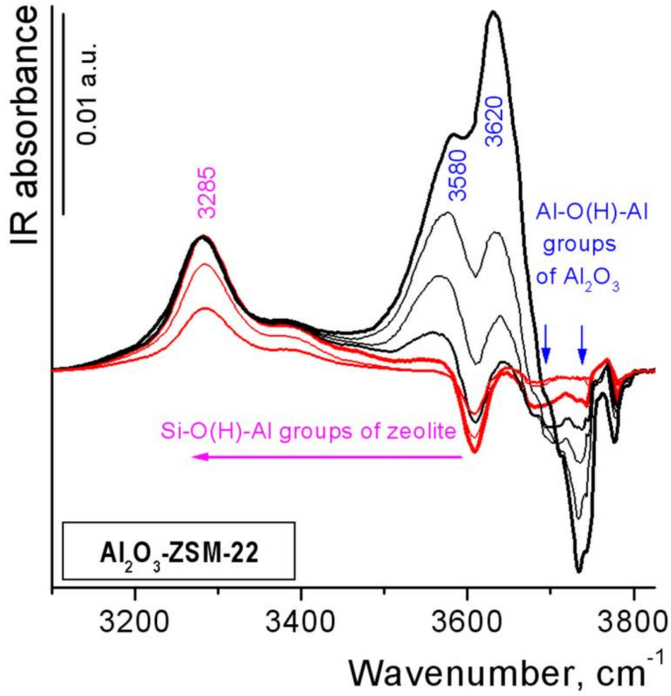

Figure S2. FTIR difference spectra of the OH stretching region during adsorption of CO on Al_2O_3-ZSM-22 support. Equilibrium CO pressures used were from 0.3 (bottom curve) to 10 mbar (top curve).
